# Functional Restriction and a New Balance between Proximal and Distal Gut: The Tools of the Real Metabolic Surgery

**DOI:** 10.1007/s11695-014-1368-x

**Published:** 2014-07-16

**Authors:** Alper Celik, Surendra Ugale

**Affiliations:** 1 Department of Metabolic Surgery, Taksim German Hospital, Siraselviler Cad. No. 119, Beyoglu Istanbul, Turkey; 2 Medical Faculty, Yeniyuzyil University, Istanbul, Turkey; 3 Department of Bariatric and Metabolic Surgery, Kirloskar Hospital, Hyderabad, India

This article aims to question the utility of current surgical methods and their alternatives for the treatment of world-threatening “diabesity” pandemic. We, as surgeons, need to address the following question honestly—“Are we offering the best surgical procedure to our patients?”

Despite the evidence suggestive of inadequate and disappointing results with restrictive techniques like vertical banded gastroplasty (VBG) and gastric banding, why is the surgical fraternity still performing restrictive operations and trying to revise the restrictive or combination operations (i.e., gastric bypass) with further restriction?

It is quite unfortunate to note the growing number of revision operations performed by bariatric and metabolic surgeons. Apparently, any restriction on humans seems to fail eventually, as observed with mechanical restrictive operations done for the surgical treatment of obesity. Therefore, we wish to highlight the potential surgical use of ileal anorexigenic neuropeptides (i.e., GLP-1, PYY, and oxyntomodulin) for the treatment of metabolic syndrome and related co-morbidities.

From this perspective, it is plausible to take a short look at the physiology of hunger and satiety. After ingesting a portion of a meal, the food is sent to the gut for digestion. Hunger is a vital primitive instinct, which is not resolved in the stomach, but in the gut. Only when food reaches the ileum does signals of satiety are released, gastric emptying slows down, and a feeling of fullness coincides. Thus, satisfaction occurs only after eating a considerable amount of food and not after the first bite. However, restrictions like a band, pouch, narrow anastomosis, or a tube, limit ingestion and are unable to provide a marked intestinal satiety as a natural consequence of the abovementioned physiological process. Mechanical restriction represents a static obstacle for the passage of food after each ingestion step, and is clearly counterintuitive from a physiological viewpoint [1]. On the other hand, ileal proximalization and activation of the ileal peptides may bring forth a type of “functional restriction” and “metabolic satiety” that limit the stocking, not ingestion.

Metabolic surgery should stay away from mechanical restriction and target a functional restriction. The only possible way to accomplish this is to activate the ileal anorexigenic neuropeptides in the early course of digestion. If the intestinal satiety signals are too weak or come late during digestion, it would be possible for an individual to consume greater amounts of food before the emergence of metabolic satiety.

Malabsorptive operations, particularly biliopancreatic diversion (BPD) and duodenal switch (DS), do have the capability to induce the release of these peptides, since the whole jejunum is bypassed, and the gastro–ileal or duodeno–ileal anastomosis deliver food directly into ileum. However, these operations have a price to pay in the form of malabsorption and nutritional deficiencies. On the other hand, “functional restriction” through surgical ileal proximalization offers a chance to release the aforementioned peptides without causing significant malabsorption. These peptides not only induce satiety, but also improve the insulinemic responses, suppress the activity and release of glucagon, and decrease endogenous glucose production and free fatty acid release.

Two major issues should be addressed though. Firstly, how can it be possible to obtain surgical ileal proximalization without causing significant malabsorption, and secondly, what is the significance of the presence of malabsorption after bariatric and metabolic surgical techniques? With regard to the latter, it is very important to realize that malabsorption itself is a disease. We all have to question the extent of the consequences of our actions on our patients in a responsible manner, since this strategy is associated with the risk of terminating of one type of addiction while triggering another. Therefore, efforts should be paid to seek for alternatives of ileal proximalization without significant malabsorption.

**Fig. 1:**
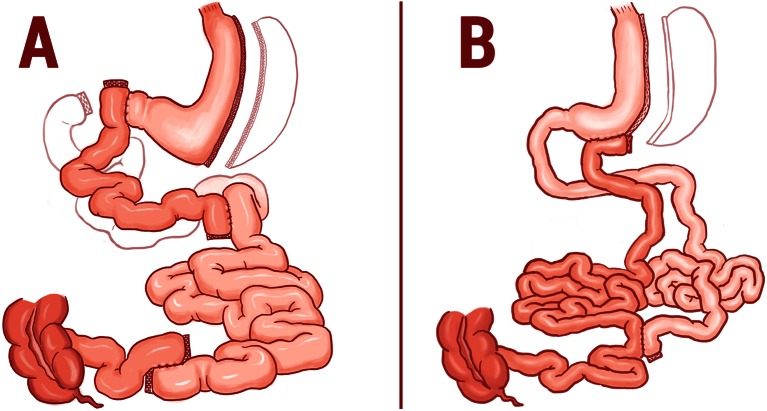
Demonstrations of ileal transposition (**a**) and transit bipartition (**b**) operations

There are two possible published surgical options that can provide a functional restriction without significant malabsorption. These are transit bipartition (TB) and ileal transposition (IT) (Fig. 1, [1, 2]).

Both operations are performed laparoscopically together with a sleeve gastrectomy in order to reduce ghrelin levels, avoid peptic ulcers, decrease the caloric intake, and avoid a gastric dilation [1]. However, these techniques use different strategies. IT maximizes distal gut activity by interposing a segment of ileum right after the stomach and minimizes proximal activity by excluding the duodenum [3, 4]. TB enhances distal activity by bringing the whole ileum to the antrum and diminishes proximal activity by shifting food from the duodenal route, which is left intact; what further minimizes malabsorption (and amplifies endoscopic access, instead of reducing it). Both procedures aim at functional (and not the mechanical) restriction, trying to avoid malabsorption, instead of having it as a beneficial goal.

In conclusion, bariatric and metabolic surgery is still evolving and there will always be a search for better surgical techniques. Novel surgical methods can potentially provide more physiological solutions both for our patients and for us as surgeons, in our battle against the pandemic of obesity and diabetes. But, that should not refrain us from questioning the appropriateness of restriction and malabsorption, and maximum effort should be made to avoid treating one condition by replacing it with a new one.
